# Chloral Poisoning

**Published:** 1884-01

**Authors:** 


### Chloral Poisoning.

What are the remedies to be employed in acute chloral poisoning? They are especially those designed (1) to sustain the action of the heart, such as ammonia and brandy; (2) to keep up the breathing by artificial respiration, if needed; (3) to keep the patient warm; and (4) to use electricity as a cutaneous stimulant. Thus far you would treat a case as an ordinary one of narcotic poisoning. But is there any remedy that will counteract the depressing effects of the chloral upon the nervous centers, and particularly the respiratory center? Yes; the remedy for this purpose is strychnia, which antagonizes the chloral. It may be used as we gave it here, hypodermically, one-sixtieth of a grain every three hours at first; and it would have been given oftener, but it was not needed. Strychnia, therefore, is indicated as the physiological antidote. It stimulates the centers which have been depressed by the chloral. When recovery takes place, it is usually rapid.

What should be the treatment of chronic chloral cases? Suppose that a patient like this says that the habit is growing upon him, and comes to you for advice, what course would you pursue? I would answer that you must reduce the dose gradually. As large doses of chloral are only given exceptionally, there will be less difficulty on this score than with opium; but as you reduce it, I would strongly advise you to give strychnia or nux vomica for its effects on the nervous system. It antagonizes the effects of the chloral, and acts as a tonic at the same time.

Those nervous centers which are reduced in their activity by the paralyzing effects of the chloral are stimulated by strychnia. If you use strychnia you may stop the chloral almost at once without any bad effects being observed. I had a case in point last summer. A gentleman who had been taking chloral for some time found himself very weak, his will-power impaired, and he felt miserable. He determined to stop off entirely. He went to Atlantic City without a single grain of chloral. He took constant out-door exercise. He was sleepless for a time, but he was able to overcome his evil habit; and, although he had been using chloral regularly for eighteen months, he recovered entirely. It should be stated, however, that while giving up the chloral habit he took, from time to time, strychnia or nux vomica.—*Philadelphia Medical Times.*

